# Predictors and Outcomes of Mental Health Conditions Among Patients with Colorectal Cancer

**DOI:** 10.1007/s12029-024-01144-1

**Published:** 2024-11-16

**Authors:** Sydney M. Taylor, Dmitry Tumin, Lance C. Tiu, Pankti S. Patel, Michael D. Honaker

**Affiliations:** 1 East Carolina University, Brody School of Medicine, Greenville, NC USA; 2 Department of Academic Affairs, East Carolina University Brody School of Medicine, Greenville, NC USA; 3 Department of General Surgery, Division of Surgical Oncology, East Carolina University Brody School of Medicine, 600 Moye Blvd, Mailstop 639, Greenville, NC 27834 USA

**Keywords:** Colorectal, Cancer, Mental health, Survival

## Abstract

**Purpose:**

Mental health (MH) conditions are common in patients with colorectal cancer (CRC) due to the unique challenges these patients encounter. The primary aim was to investigate predictors of new onset MH conditions after a diagnosis of CRC and determine the association of new MH conditions on survival.

**Methods:**

A single institution, retrospective study was conducted. A multivariable Fine-Gray competing risks model was used to describe the primary study outcome of new MH diagnosis in patients at least 18 years of age with CRC. Survival was modeled using Cox proportional hazards regression with a time-varying covariate for new MH diagnosis.

**Results:**

456 patients were identified for inclusion, with 16% developing a new MH condition and 29% dying during follow-up. A new MH condition was more likely among non-Hispanic white patients compared to non-Hispanic black and were less likely among those who are male or had a pre-cancer MH condition. The onset of a new MH condition was associated with a threefold decrease in survival. In addition, having a pre-cancer MH condition decreased survival nearly twofold.

**Conclusions:**

Our findings emphasize the importance of new-onset MH in patients after CRC diagnosis. Standardized screenings may alleviate some of the MH burden that patients with CRC experience in addition to potentially improving the overall health of patients.

Implications for Cancer Survivors.

MH conditions may impact not only CRC outcomes but may direct future studies analyzing the risks of new onset MH conditions in other types of cancers, further expanding the importance of psychiatric support in patients with cancer.

## Introduction

Colorectal cancer (CRC) is the third most common cause of cancer-related mortality in the USA [1]. Patients with colorectal cancer experience significant psychosocial distress [2]. Mental illness and comorbid health conditions are often intertwined with a cancer diagnosis, and patients with pre-existing mental illness may face an overall worse prognosis with possible increased mortality [3–6].

The onset of new mental health (MH) conditions among patients with CRC is especially common among patients who have additional medical comorbidities, post-cancer complications, or secondary to the cancer operation itself (e.g., the need for a stoma) [6]. MH conditions present at the time of diagnosis or reported after diagnosis among patients with colorectal cancer may be associated with increased mortality, due to their negative impact on treatment adherence and completion [7].

In this study, we investigated predictors of new onset MH conditions after a diagnosis of CRC and sought to determine if the onset of new MH conditions is associated with decreased overall survival. We hypothesized that the onset of new MH conditions after colorectal cancer diagnosis will lead to decreased rates of survival independent of the presence of previously existing MH conditions.

## Methods

### Data Source and Study Population

A single institution, retrospective observational study was conducted at ECU Health Medical (ECUHMC) Center in Greenville, NC, USA. ECUHMC, together with 8 affiliated hospitals, serves the predominately rural eastern region of the state. Patients at least 18 years of age with a first diagnosis of CRC from 2017 through 2020 were identified from the institutional tumor registry. Patients with missing data on study variables were excluded from the analysis. Among eligible patients, entry into the study was defined based on the earliest CRC diagnosis during the study period, and any subsequent new diagnosis of colorectal cancer was treated as a censoring event, such that follow-up after a subsequent CRC diagnosis did not confound the findings from our study. Surveillance was performed as per the National Comprehensive Cancer Guidelines for colon and rectal cancer[8, 9]. The results are reported per the Strengthening the Reporting of Observational Studies in Epidemiology (STROBE) guidelines [10].

### Variables and Outcomes

The primary outcome was a dichotomous measure of any new MH condition after CRC diagnosis. Data on new MH conditions after a diagnosis of CRC were obtained from the electronic medical record. Assessment of MH during subsequent oncology follow-up was not standardized; routine practice at ECUHMC consists of obtaining a depression screen and distress thermometer. Patients meeting criteria are offered referral to a mental health professional for counseling. We identified the first new diagnosis of a MH condition after the diagnosis of colorectal cancer, based on the earliest mention of a new MH condition or its treatment in the medical record, not including recurrence of previously diagnosed MH conditions. We considered the onset of a new MH condition to be the documented date of diagnosis if this information was available from the medical record. Otherwise, we defined the onset of a new MH condition based on the earliest mention of a new MH condition in the medical record. The lead author, in consultation with the study team, developed criteria for collection of MH conditions. Many of these conditions included states such as anxiety or depression, but did not have to formally see a psychiatrist for these diagnoses. These data were extracted by a single investigator to maintain consistency.

The secondary outcome was overall patient survival. Overall survival and date of combined last patient contact (i.e., date of death or date of censoring) were obtained from the institutional tumor registry, with original data collection being based on guidelines for the American College of Surgeons [11]. Follow-up data were queried from the tumor registry through August 2023. Other variables collected for this study included presence of any pre-existing MH diagnoses; sex; age at diagnosis; race and ethnicity (non-Hispanic white (NHW), non-Hispanic black (NHB), or other); tobacco use history (current, previous, or none); primary payer at the time of cancer diagnosis (Medicare, Medicaid, private, uninsured, or military insurance); cancer stage (I–IV), cancer site (colon or rectum); marital status at time of diagnosis (married; divorced, separated, or widowed; or single); and comorbidities, including coronary artery disease (CAD), congestive heart failure (CHF), chronic obstructive pulmonary disease (COPD), connective tissue disease, and diabetes mellitus. We also controlled for rural vs. urban residence at the time of data extraction, based on the United States Division of Agriculture-Economic Research Service urban influence codes [12].

### Statistical Analysis

Categorical data were summarized using percentages, while continuous data were summarized as medians with interquartile ranges (IQR). In bivariate analysis, we compared study variables based on whether patients had any new mental health diagnoses using Chi-square or Fisher’s exact tests for categorical variables, and Wilcoxon rank-sum tests for continuous variables. A cumulative incidence function was used to describe the primary study outcome of a new MH condition, and we used a multivariable Fine-Gray competing risks model to generate sub-hazard ratios (SHR) for each independent variable predicting new mental health diagnosis. The secondary outcome of survival was described using a Kaplan–Meier plot and analyzed further using multivariable Cox proportional hazards regression with a piecewise-constant time-varying covariate representing the change in mortality hazard after diagnosis of a new MH condition. Data analysis was completed using Stata/SE 18.0 (College Station, TX, USA: StataCorp, LP), and *P*-value < 0.05 was considered statistically significant.

## Results

**Fig. 1:**
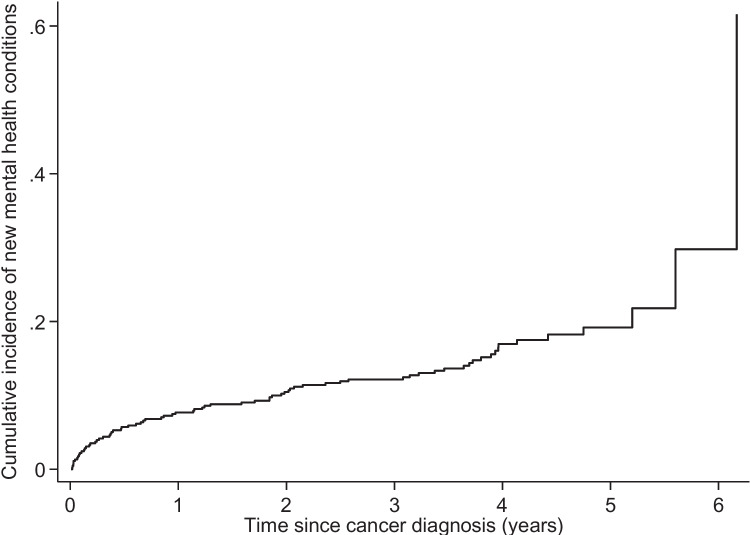
Cumulative incidence function of new mental health diagnosis after cancer diagnosis

We identified 482 patients with eligible cancer diagnoses during the study period, of whom we excluded 2 cases with incomplete survival data and 24 cases with missing data on other study variables. Among the remaining sample (*N* = 456, 50%/50% male/female, median age 66 years), 73 patients developed a new MH condition after cancer diagnosis (16%). Among patients who developed a new MH condition, median time from cancer diagnosis until documentation of the new MH condition was 14 months (IQR 3, 37; Fig. 1).

**Fig. 2:**
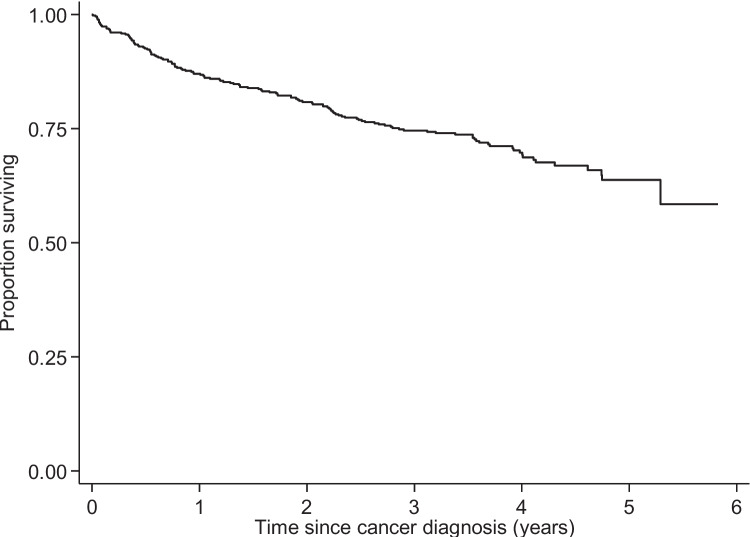
Kaplan–Meier plot of patient survival after cancer diagnosis

Overall, 133 patients died (29%) during follow-up, and median follow-up time for survival analysis was 3 years (IQR 2, 4; Fig. 2).

Patient characteristics are compared based on onset of a new MH condition in Table 1.

**Table 1:** Patient characteristics by onset of new mental health condition after cancer diagnosis (*N* = 456)

Variable	No new mental health condition (*N* = 383)	New mental health condition (*N* = 73)	*P*
	*N* (%) or median (IQR)	*N* (%) or median (IQR)	
Pre-cancer mental health diagnosis	78 (20%)	9 (12%)	0.109
Sex			0.067
Female	186 (49%)	44 (60%)	
Male	197 (51%)	29 (39%)	
Age at cancer diagnosis (years)	66 (56, 75)	68 (56, 77)	0.710
Race and ethnicity			0.032
Non-Hispanic White	230 (60%)	53 (73%)	
Non-Hispanic Black	140 (37%)	16 (22%)	
None of the above	13 (3%)	4 (5%)	
Tobacco use			0.839
Current	63 (16%)	13 (18%)	
Previous	145 (38%)	25 (34%)	
None	175 (46%)	35 (48%)	
Primary insurance coverage			0.420
Medicare	203 (53%)	38 (52%)	
Medicaid	28 (7%)	9 (12%)	
Private	123 (32%)	20 (27%)	
Uninsured	21 (5%)	3 (4%)	
Military insurance	8 (2%)	3 (4%)	
Cancer stage			0.272
I	93 (24%)	18 (25%)	
II	112 (29%)	17 (23%)	
III	118 (31%)	20 (27%)	
IV	60 (16%)	18 (25%)	
Cancer site			0.775
Colon	320 (84%)	60 (82%)	
Rectum	63 (16%)	13 (18%)	
County of residence			0.842
Urban or suburban	178 (46%)	33 (45%)	
Rural	205 (54%)	40 (55%)	
Marital status			0.225
Married	189 (49%)	28 (38%)	
Divorced, separated, or widowed	118 (31%)	27 (37%)	
Single	76 (20%)	18 (25%)	
CAD	38 (10%)	14 (19%)	0.023
CHF	19 (5%)	5 (7%)	0.508
COPD	26 (7%)	5 (7%)	0.985
Connective tissue disease	9 (2%)	5 (7%)	0.041
Diabetes	92 (24%)	19 (26%)	0.714

*IQR*, interquartile range; *CAD*, coronary artery disease; *CHF*, congestive heart failure; *COPD*, chronic obstructive pulmonary disease

Patients diagnosed with new MH conditions were more likely to being NHW. The multivariable model of new MH condition onset is shown in Table 2.

**Table 2:** Multivariable competing-risks regression model of onset of new mental health condition after cancer diagnosis (*N* = 456)

Variable	SHR	95% CI	*P*
Pre-cancer mental health diagnosis	0.4	0.2, 0.9	0.026
Sex			
Female	Ref		
Male	0.6	0.4, 0.99	0.043
Age at cancer diagnosis (years)	1.0	0.9, 1.0	0.276
Race and ethnicity			
Non-Hispanic White	Ref		
Non-Hispanic Black	0.4	0.2, 0.6	0.001
None of the above	1.3	0.4, 3.8	0.682
Tobacco use			
Current	Ref		
Previous	1.0	0.5, 2.0	0.943
None	1.0	0.5, 2.0	0.940
Primary insurance coverage			
Medicare	Ref		
Medicaid	1.2	0.4, 3.4	0.768
Private	0.7	0.3, 1.7	0.466
Uninsured	0.6	0.2, 1.9	0.369
Military insurance	2.0	0.6, 7.5	0.284
Cancer stage			
I	Ref		
II	0.8	0.4, 1.5	0.420
III	0.8	0.4, 1.6	0.550
IV	1.0	0.7, 2.6	0.411
Cancer site			
Colon	Ref		
Rectum	1.0	0.6, 2.0	0.709
County of residence			
Urban or suburban	Ref		
Rural	1.0	0.7, 1.7	0.784
Marital status			
Married	Ref		
Divorced, separated, or widowed	1.5	0.8, 2.6	0.189
Single	1.7	1.0, 3.3	0.095
CAD	2.0	1.0, 3.8	0.046
CHF	1.5	0.5, 4.3	0.501
COPD	0.7	0.3, 2.0	0.537
Connective tissue disease	3.6	1.5, 8.6	0.005
Diabetes	1.0	0.5, 1.7	0.814

*CI*, confidence interval; *Ref*., reference; *SHR*, subhazard ratio; *CAD*, coronary artery disease; *CHF*, congestive heart failure; *COPD*, chronic obstructive pulmonary disease

A new MH condition was more likely among patients who were NHW compared to NHB and were less likely among those who are male or had a pre-cancer MH condition. Additional risk factors for the development of a new MH condition include having CAD and connective tissue disease.

The multivariable Cox proportional hazards model of patient survival is shown in Table 3.

**Table 3:** Multivariable Cox proportional hazards model of patient survival (*N* = 456)

Variable	HR	95% CI	*P*
Onset of new mental health condition	3.3	2.0, 5.3	< 0.001
Pre-cancer mental health diagnosis	1.9	1.2, 2.9	0.006
Sex			
Female	Ref		
Male	1.1	0.8, 1.7	0.514
Age at cancer diagnosis (years)	1.05	1.02, 1.07	< 0.001
Race and ethnicity			
Non-Hispanic White	Ref		
Non-Hispanic Black	1.1	0.7, 1.7	0.597
None of the above	0.9	0.3, 2.9	0.850
Tobacco use			
Current	Ref		
Previous	0.8	0.4, 1.4	0.368
None	0.5	0.3, 0.9	0.024
Primary insurance coverage			
Medicare	Ref		
Medicaid	1.3	0.6, 3.0	0.512
Private	1.4	0.8, 2.4	0.244
Uninsured	1.4	0.5, 4.1	0.541
Military insurance	1.0	0.3, 3.2	0.967
Cancer stage			
I	Ref		
II	1.7	0.9, 3.5	0.123
III	3.2	1.7, 6.0	< 0.001
IV	13.5	7.2, 25.1	< 0.001
Cancer site			
Colon	Ref		
Rectum	0.9	0.5, 1.5	0.589
County of residence			
Urban or suburban	Ref		
Rural	0.9	0.6, 1.4	0.739
Marital status			
Married	Ref		
Divorced, separated, or widowed	1.5	0.9, 2.3	0.086
Single	1.8	1.1, 3.0	0.031
CAD	1.1	0.7, 1.9	0.627
CHF	2.5	1.3, 5.0	0.009
COPD	1.1	0.6, 1.9	0.875
Connective tissue disease	1.1	0.4, 2.9	0.878
Diabetes	1.0	0.7, 1.6	0.914

*CI*, confidence interval; *HR*, hazard ratio; *Ref*., reference; *CAD*, coronary artery disease; *CHF*, congestive heart failure; *COPD*, chronic obstructive pulmonary disease

The onset of a new MH condition was associated with a threefold increase in mortality hazard (hazard ratio [HR]: 3.3; 95% CI: 2.0, 5.3; *P* < 0.001). In addition, having a pre-cancer MH diagnosis increased mortality hazard nearly twofold. Older age at cancer diagnosis, current tobacco use, patients with cancer stage III or IV, and being single were associated also with an increase in mortality hazard.

Of individuals with any mental health diagnosis both before or after a CRC diagnosis, approximately 97% of patients had either anxiety or depression with 3% having other mental health conditions.

## Discussion

While depression has been widely studied in patients with cancer, analysis distinguishing MH conditions before are compared to after a diagnosis of cancer is limited within the literature. In this study of over 450 patients, a pre-existing MH condition was found to be protective against the development of a new MH condition after CRC diagnosis. However, a pre-existing MH condition resulted in a twofold decrease in overall survival, while the onset of a new MH condition after CRC diagnosis (identified in 16% of the cohort) was associated with a threefold decrease in overall survival.

More advanced stage of disease is a risk factor for the development of anxiety and depression in patients diagnosed with GI cancers [13, 14]. This contrasts with our findings, where cancer stage was not a significant risk factor for the development of new MH conditions. This raises the question of what serves as the main driver for the development of new MH conditions. Our finding may represent the challenging treatment, stigma, or emotional burden of patients across all stages, with MH conditions affecting individuals regardless of cancer stage. Tracking MH conditions among patients with cancer is also challenging. There is a lack of standardized protocols, and the initiative often falls upon the treating physicians, who are already restricted by time constraints [15]. Interdisciplinary teams consisting of oncologists, psychiatrists, clinical psychologists, and social workers have been successful at identifying and treating cancer patients with a MH condition [16]. Thus, integrating MH support for patients at time of diagnosis, and throughout their treatment and surveillance periods may improve the patient’s overall mental health.

In the current study, NHW patients had a greater risk of developing MH conditions than patients from other racial and ethnic groups. This could be explained by structural barriers to mental health care access preventing NHB patients from receiving care and thus not receiving evaluation for MH conditions. In addition, a higher degree of stigma around MH may discourage seeking care among this population [17]. MH support may require the expertise of a specialty healthcare provider, which can be difficult to obtain for patients with limited access to healthcare or other socioeconomic factors that impede the ability to obtain MH treatment. Additionally, female patients were more likely to develop a new MH condition after CRC diagnosis than male patients. This finding is consistent with previous research, with a possible explanation being that female patients may be more sensitive to hormone fluctuations associated with CRC, which may then affect serotonergic/noradrenergic processes involved in mood [18]. Another plausible explanation may be that female patients are more willing to report symptoms of MH concerns and subsequently seek care for these concerns than male patients.

Additional risk factors for the development of new MH conditions include CAD and connective tissue disease. This may be attributed to the physical burden on one’s body with these diagnoses through medications and symptomatology, and when compounded with a cancer diagnosis, increases the likelihood of worsening mental health.

Although patients with pre-existing MH conditions had increased risk of mortality, they were less likely to develop a new MH condition. This finding may be secondary to familiarity with and established care for patients’ pre-existing MH conditions, and these patients’ ongoing use of treatment, interventions, or coping mechanisms that are protective from developing an additional MH condition. Others have shown lower quality of life, low perceived social support, maladaptive coping style, and low spirituality to be associated with an increased risk of experiencing a new MH diagnosis after a cancer diagnosis [19].

Previous studies examining mortality have focused on pre-existing MH conditions, or primarily examined anxiety and depression as risk factors for mortality in CRC patients [18, 20]. Our results demonstrated that development of a MH condition after a CRC diagnosis is associated with a threefold increase in mortality. This may represent decreased support in cancer care, as evinced by the association of decreased survival with divorced, separated, or widowed marital status. Other possible explanations include decreased adherence to treatment, opting out of support programs, or being diagnosed at a more advanced stage [21, 22], although stage was not independently associated with increased mortality in the current study.

Additional explanations for these findings include the notion that poor MH, when unremitting, may be tied to suppression of the immune system through the hypothalamic–pituitary–adrenal axis. As a result, tumor surveillance in the body may be directly impacted, leading to worse cancer outcomes [18]. The finding of increased mortality associated with a new onset MH condition may also be attributed to the specific type of MH diagnosis. For example, substance use disorder can lead to physical deterioration, or specific diagnoses like schizophrenia or psychosis with associated poor self-care can result in worsening physical health when combined with CRC treatment [23].

There are several limitations to the present study. The retrospective, single-institution design is limited in its generalizability to other populations. In addition, chart review is susceptible to human error, although one reviewer obtained MH conditions for all patients to assure internal validity. While many of the MH conditions in our cohort may have been diagnosed formally with Diagnostic and Statistical Manual of Mental Disorders (DSM)-V criteria in mind, this was not an inclusion criterion for MH diagnosis in our study, as the majority of patients were not diagnosed by a MH specialist. Additionally, because of the relatively low numbers of some MH conditions, we were not able to determine which specific conditions had the greatest impact on study outcomes. Lastly, not all physicians standardized MH follow-up or screening in their appointments post-cancer diagnosis, which could lead to underreporting of patients’ MH concerns. Due to this, we also did not track if patients in our study received mental health treatment.

## Conclusion

Poor MH was a predictor for decreased survival when compounded with a cancer CRC diagnosis. Given that MH conditions are common in this population, the finding of increased mortality associated with new MH conditions as well as existing diagnoses may profoundly impact the treatment course for CRC. The current study provides support for MH screening programs in patients diagnosed with CRC. Improving awareness and treatment of MH conditions arising in conjunction with CRC may lead to improved oncologic outcomes. Future studies should aim at implementing strategies for prevention, early diagnosis, and treatment of MH conditions.

## Data Availability

Data that support the findings of this study have been deposited in the Figshare Repository 10.6084/m9.figshare.26510908.

## Declarations

The authors declare that no funds, grants, or other support were received during preparation of this manuscript. DT discloses salary support from Kate B. Reynolds Charitable Trust Foundation and Lilly Grant Office for research and quality improvement projects unrelated to the present work.
